# The complete chloroplast genome sequence of *Actinidia chinensis* Planch. ‘Hongyang’, a typical red core pulp in China

**DOI:** 10.1080/23802359.2022.2057249

**Published:** 2022-04-01

**Authors:** Xia Liu, Chong Sun, Mingzhi Li, Jing Liu, Wenlin Zhang, Ran Liu, Yiqing Liu, Youzhang Le, Jianbin Lan

**Affiliations:** a College of Landscape Architecture and Life Science, Chongqing University of Arts and Sciences, Chongqing, China; b College of Horticulture and Landscape Architecture, Southwest University,Chongqing, China; c College of Horticulture and Landscape Architecture/Chongqing Key Laboratory of Economic Plant Biotechnology, Yangtze University, Hubei, China; d Bio&Data Biotechnologies Co. Ltd, Guangzhou, China; e Chongqing Fuyuan Agricultural Biotechnology Research Institute, Chongqing, China; f Wuhan Academy of Agricultural Sciences, Hubei, China

**Keywords:** *Actinidia chinensis* Planch. ‘Hongyang’, chloroplast genomes, Actinidiaceae, phylogenetic analysis

## Abstract

*Actinidia chinensis* Planch. ‘Hongyang’ Wu and Li 1993, also known as red-fleshed kiwifruit, has a high vitamin C content and with high economic and nutritional value. Here, we assembled the complete chloroplast genome of *A. chinensis* Planch. ‘Hongyang’, which was 156,267 bp in length, contained a large single-copy region (LSC) of 87,866 bp, a small single-copy region (SSC) of 20,335 bp, and two inverted repeat (IR) regions of 24,033 bp. In addition, the chloroplast genome contained 132 genes, including 85 protein-coding, 39 tRNA, and eight rRNA genes. Overall GC content in the genome was 37.2%, with the corresponding values in the LSC, SSC, and IR regions of 35.5%, 31.1%, and 42.9%, respectively. Phylogenetic analysis indicated that *A. chinensis* Planch. ‘Hongyang’ was clustered with that of *A. callosa* var. *strigillosa*, *A. deliciosa*, *A. melanandra*, *A. chinensis* and *A. setosa* in the same branch.

In 1993, Wu first published *Actinidia chinensis* ‘Hongyang’ Wu as a new cultivar (Wu and Li 1993). The new cultivar is a dioecious and deciduous plant native to Sichuan, China (Wang et al. 2003). This cultivar approved by Sichuan Province Crop Variety Certification Committee in China, and was officially named in 1997 (Ye 2004). It is an important germplasm resource in the Actinidiaceae family and is widely cultivated throughout China. Cross-section of the fruit shows red lines radially distributed along the core, hence the name ‘Red Sun’ kiwifruit (Li 2004). The fruit is short and cylindrical, the average fruit weight is 60–110 g, the maximum fruit weight is 130 g, and vitamin C contained varied from 135.77 to 250 mg/100 g fresh weight (Ye 2004; Wang 2013). Therefore, it is considered a nutritionally and medicinally valuable fruit (Hemalatha et al. 2020). The availability of the *A. chinensis* ‘Hongyang’ chloroplast genome will greatly contribute to species identification, phylogenetic analysis, and genetic engineering study of the family Actinidiaceae.

Fresh *A. chinensis* ‘Hongyang’ leaves were collected from an agriculture demonstration park in Chongqing, China (29.2497 N, 105.8873 E). A voucher specimen was also deposited at the Chongqing University of Arts and Sciences Herbarium (HY1) under accession number CUAS-Hymh01 (Jianbin Lan, 279139722@qq.com). Total DNA was isolated using a modified CTAB method (Doyle and Doyle 1987). The DNA library was sequenced by Hefei Bio&Data Biotechnologies Inc. (Hefei, China) on the BGISEQ-500 platform with 150 paired-end reads. In total, 34.3 million high-quality clean reads were generated with adaptors trimmed. Then, SPAdes Assembler v3.9.0 (Bankevich et al. 2012) was used for de novo assembly with *A. chinensis* (GenBank accession MT712168) as a reference. The chloroplast genome was annotated using CpGAVAS (Liu et al. 2012) and GeSeq software (Tillich et al. 2017). Annotation errors were corrected manually. The complete chloroplast genome sequence of *A. chinensis* ‘Hongyang’ was submitted to GenBank under the accession number of MW596240.

The circular chloroplast genome of *A. chinensis* ‘Hongyang’ with a length of 156,267 bp and contained two inverted repeat (IRa and IRb) regions of 24,033 bp, which were separated by a large single copy (LSC) region of 87,866 bp, and a small single copy (SSC) region of 20,335 bp. The chloroplast genome contains 132 genes, including 85 protein-coding genes, 39 tRNA genes, and 8 rRNA genes. Among the genes, 18 genes were duplicated in the IR regions. In total, 19 genes contained two exons and three genes (*ycf3* and two *rps12*) with three exons. The overall GC content of *A. chinensis* ‘Hongyang’ was 37.2%, and the corresponding values in LSC, SSC and IR regions were 35.5%, 31.1%, and 42.9%, respectively. GC content of IRs region was the highest. Among the genes, 8 protein coding, 7 tRNA, and 4rRNA genes were found duplicated in IR regions. Compared with the published chloroplast genome of *A. chinensis* (MT712168), *A. chinensis* ‘Hongyang’ contained 172 mutation sites, including 47 indel sites, 122 SNP sites and 3 substitution sites (GAA/TTC, ATAAA/TAGTT, AT/TA). Among them, there were 120 LSC and 8 IRB/IRA areas, respectively (16 in total), and 37 SSC areas.

To analyze the phylogenetic relationships between *A. chinensis* ‘Hongyang’ and other members of the genus *Actinidia*, we used the complete chloroplast genome sequences of 23 species, and constructed a phylogenetic tree (Figure 1). These sequences were aligned with MAFFT v7.407 (Katoh and Standley 2013). A maximum -likelihood (ML) tree was performed with RAxML v8 (Alexandros 2014) using 1000 bootstrap. Results showed that *A. chinensis* ‘Hongyang’ was clustered with that of *A. callosa* var. *strigillosa*, *A. deliciosa*, *A. melanandra*, *A. chinensis* and *A. setosa* in the same branch, and with bootstrap support values of 100%.

**Figure 1.**
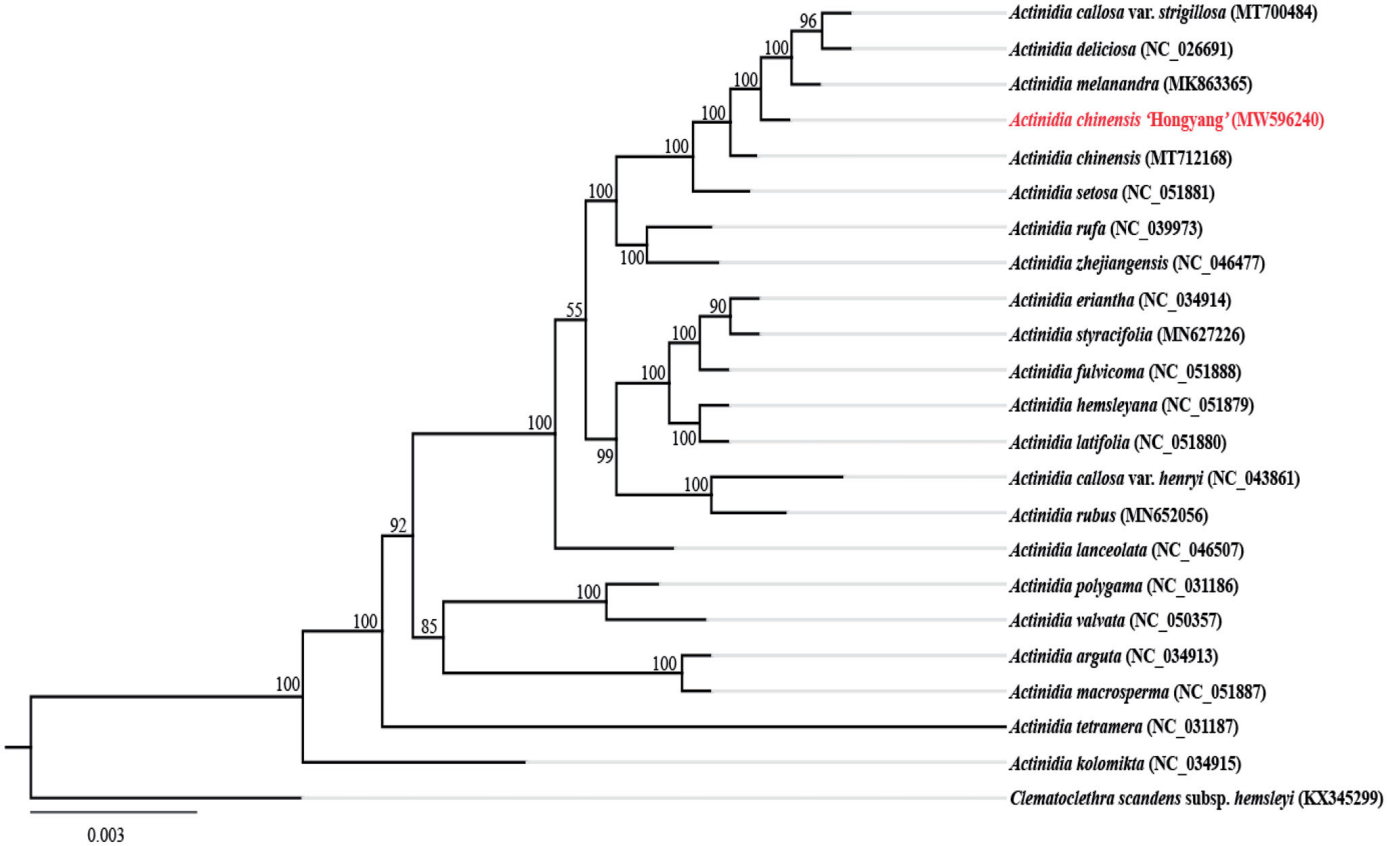
Maximum-likelihood phylogenetic tree of *Actinidia chinensis* ‘Hongyang’ and other related species based on the complete chloroplast genome sequences. Number on each node indicates bootstrap support value.

## Ethical statement

This research does not involve ethical research. The collection of plant material carried out in accordance with guidelines provided by the authors’ institution (the Chongqing University of Arts and Sciences) and national.

## Author contributions statement

Xia Liu and Yiqing Liu designed the study, writing and revised the manuscript; Chong Sun, Mingzhi Li, Jing Liu, and Wenlin Zhang involved in the process of sequences editing and phylogenetic analyses; Youzhang Le, Jianbin Lan and Ran Liu participated in the collection and identification of plant material. All authors read and approved the final manuscript, and agreed to be accountable for all aspects of the work.

## Data Availability

The genome sequence data that support the findings of this study are openly available in GenBank of NCBI at (https://www.ncbi.nlm.nih.gov/) under the accession no. MW596240. The associated BioProject, SRA, and Bio-Sample numbers are PRJNA701792, SRR13708187, and SAMN17911693, respectively.
